# Chia seeds ameliorate cardiac disease risk factors via alleviating oxidative stress and inflammation in rats fed high-fat diet

**DOI:** 10.1038/s41598-023-41370-4

**Published:** 2024-02-05

**Authors:** Mohamed Aref, Eman Mahmoud FaragAllah, Nehal I. A. Goda, Mohammed H. Abu-Alghayth, Mosleh M. Abomughaid, Heba H. Mahboub, Khairiah Mubarak Alwutayd, Hadeel A. Elsherbini

**Affiliations:** 1 Anatomy and Embryology Department, Faculty of Veterinary Medicine, Zagazig University, Zagazig, 44511 Sharkia Egypt; 2 Physiology Department, Faculty of Medicine, Zagazig University, Zagazig, Egypt; 3 Department of Histology and Cytology, Faculty of Veterinary Medicine, Zagazig University, Zagazig, 44511 Egypt; 4 Department of Medical Laboratory Sciences, College of Applied Medical Sciences, University of Bisha, 255, Al Nakhil, 67714 Bisha, Saudi Arabia; 5 Department of Aquatic Animal Medicine, Faculty of Veterinary Medicine, Zagazig University, Zagazig, Sharkia Egypt; 6 Department of Biology, College of Science, Princess Nourah bint Abdulrahman University, P.O. Box 84428, 11671 Riyadh, Saudi Arabia

**Keywords:** Biochemistry, Anatomy, Biomarkers, Cardiology, Health care, Medical research, Risk factors

## Abstract

Obesity upsurges the risk of developing cardiovascular disease, primarily heart failure and coronary heart disease. Chia seeds have a high concentration of dietary fiber and increased concentrations of anti-inflammatoryand antioxidant compounds. They are used for weight loss plus enhancing blood glucose and lipid profile. The current perspective was commenced to examine the protective influence of chia seeds ingestion on cardiovascular disease risk factors in high-fat diet-fed rats. Forty male albino rats (with an initial body weight of 180–200 g) were used in this study. Rats were randomly and equally divided into 4 groups: Group I was the control group and group II was a control group with chia seeds supplementation. Group III was a high-fat diet group (HFD) that received HFD for 10 weeks and group IV was fed on HFD plus chia seeds for 10 weeks. In all groups Echocardiographic measurements were performed, initial and final BMI, serum glucose, AC/TC ratio, lipid profile, insulin (with a computed HOMA-IR), creatinine phosphokinase-muscle/brain (CPK-MB), CRP, and cardiac troponin I (cTnI) and MAP were estimated. Whole heart weight (WHW) was calculated, and then WHW/body weight (BW) ratio was estimated. Eventually, a histopathological picture of cardiac tissues was performed to assess the changes in the structure of the heart under Haematoxylin and Eosin and Crossmon’s trichrome stain. Ingestion of a high diet for 10 weeks induced a clear elevation in BMI, AC/ TC, insulin resistance, hyperlipidemia, CRP, CPK-MB, and cTnI in all HFD groups. Moreover, there was a significant increase in MAP, left ventricular end diastolic diameter (LVEDD), and left ventricular end systolic diameter (LVESD). Furthermore, histological cardiac examination showed structural alteration of the normal structure of the heart tissue with an increase in collagen deposition. Also, the Bcl-2 expression in the heart muscle was significantly lower, but Bax expression was significantly higher. Chia seeds ingestion combined with HFD noticeably ameliorated the previously-recorded biochemical biomarkers, hemodynamic and echocardiography measures, and histopathological changes. Outcomes of this report reveal that obesity is a hazard factor for cardiovascular disease and chia seeds could be a good candidate for cardiovascular system protection.

## Introduction

Obesity causes at least 2.8 million deaths every year due to its associated comorbidities and the global prevalence of obesity is nearly tripled between 1975 and 2016^1^. Obesity is a crucial risk factor for cardiovascular diseases (CVD)^2^. This could be attributed to obesity itself or because of linked medical conditions such as diabetes, hypertension, and insulin resistance^3,4^. Obesity plays a substantial role in atherosclerosis^5^ and coronary artery disease^6^. It also induces functional and structural alterations of the heart, which in turn leads to heart failure^7^. The changed myocardial structure elevates the danger of atrial fibrillation^8^ and sudden cardiac death^9^.

Apoptosis and cardiomyocyte programmed cell death have a pivotal role in obesity-induced heart disease development and progression^10^. Accumulation of triglyceride in the heart muscle in obese patients enables the release of toxic like diacylglycerol and ceramide, producing apoptosis of cardiomyocytes^11^.

An essential population behavior is a potent tendency to utilize foods, nutritional supplements, or diets that have reporting of weight loss^12,13^. Also, the use of dietary fibers enhances the loss of weight, improves levels of lipid and blood glucose, and lessens blood pressure^14,15^.

Chia is a herbal extract that belongs to the family Lamiaceae^16^. It has been discovered by ancient Central American civilization to use chia as a food and medicinal source, and more recently it has been extensively used by researchers owing to its different chemical constituents that give essential health advantages to individuals consume it^17,18^.

Several studies reveal that chia seeds are rich in lipids, protein,soluble and insoluble dietary fibers,and phenolic compounds. Such reports demonstrate that consumption of chia seeds can reduce levels of triglyceride and cholesterol,control good glycemic levels, help in weight loss, and lessen risk factors associated with chronic diseases such as Type 2 Diabetes Mellitus (T2DM),obesity,lessening postprandial glucose excursion^19–23^,and inflammation^24^.A recent report documents that chia seeds carry omega-3 fatty acids and antioxidants which are essential components in minimizing the risk of CVD^25^.

Hence, the main goal of this report is to assess the potential cardio-protective effects of chia seeds in rats that received high-fat diets via assessing biochemical-hemodynamic biomarkers and illustrating anatomical and histopathological pictures.

## Material and methods

This study was carried out on 40 adult male albino rats (average body weight 180-200 g). During the first 14 days of acclimatization, the animals were fed a regular rodent diet. After this period, rats were randomly distributed into four groups of 10 animals per group. The first group was a control (group I) and the second one was a control and dietary supplement in chia seeds (group II). The third group was dietary supplemented with a High-fat diet (group III) and the last group received a high-fat diet with chia seeds supplementation (group IV).Groups I and II were fed on standard chow (12.6 kJ/g) which was composed of 18 % protein, 77% carbohydrate, and 5% fat. While groups III and IV were enriched ina high-fat diet (HFD) (18.4% protein,60.3% fat, and 21.3% carbohydrate)as described by^26^ for 10 weeks to induce chronic obesity.Chia seeds *(Salvia Hispanica*),obtained from Holland & Barrett Company, Leicester, UK,were represented 15% of diet weight in groups supplemented with chia^27^.This percentage was considered a quantity that theoretically contains enough omega 3 (ω-3) and alpha-linolenic acid (ALA)^28^.

The death percentage during the whole study was 3 %.The experiment was performed following the principles for the use and care of research animals and was accepted by the Faculty of Medicine, Zagazig University under supervision of the Institutional Research Board (ZU-IACUC/3/F/249/2022).

### Anthropometric measures

Calculating body mass index (BMI) and abdominal circumference (AC) / thoracic circumference (TC) ratio:

Body mass index (BMI) represents body weight (gm) / length2 (cm^2^), this calculation could be utilized as an indicator for obesity where the value of cutoff of obesity BMI is exceeding 0.68 gm/cm^2^^29^. To estimate AC/TC ratio, we did a division for AC by TC which is considered an indicator of the development of visceral obesity^29^.

### Assessment of hemodynamic biomarkers

Hemodynamic parameters including systolic, diastolic, heart rate, and mean arterial blood pressure,) were computed using the Recorder (Non-Invasive Blood Pressure) using rat tail-cuff protocol (Kent Scientific Corporation, Connecticut, Torrington, USA)^30^.

### Echocardiographic assessment

After 2 weeks(after fasting for 12hrs), the anesthesia was carried out for animals via injection of urethane intraperitoneally(1200mg/kg)^31^.Following an-anesthetizing animals, every rat was shaved with precautions from the chest wall, and then rats were fastened in a supine position with stretching front legs. Following that, ultrasound gel was added to the pericardium.

Echocardiography in the transthoracic cavity was performed using a 7.5MHz transducer and a GE ultrasonography. Images were taken from the heart in two-dimensional (2-D) mode. The M-mode line was put in a perpendicular way to the interventricular septum, then gointo the structures of the Left Ventricle (LV), at the level of the chordae tendinea, below the Mitral valve, and images (M-mode)were caught^32^.

These biomarkers were computed: Left Ventricular End Systolic Diameter (LVESD) and Ventricular End Diastolic Diameter (LVEDD)according to^33^.

### Blood collection

Collection of blood samples was carried out (at the end of the experiment) after overnight fasting and calculation of blood pressure, then, hematological samples were collected from the orbital sinus vein of each rat after ether inhalation^34^ for measuring serum Biochemical parameters.

### Measurement of serum biochemical indices

#### Serum glucose level

The level of serum glucose was estimated by using glucose enzymatic (GOD-PAP)-liquizyme Kits (Biotechnology, Egypt), according to^35^.

#### Measurement of serum insulin level

The level of serum insulin was estimated via using rat insulin enzyme-linked immunosorbent assay kitBioSource Europe S.A.-Rue de l’Industrie, 4-A- 1300 Nivelles-Belgium according to^36^.

For estimating of homeostasis model for insulin resistance (HOMA-IR): This equation was applied; [insulin (μU/mL) x glucose (mg/dl) /405]^37^.

#### Assessment of serum total cholesterol (TC) level

The TC level was measured following^35^using rat cholesterol enzyme-linked immunosorbent assay kit, (BioSource Europe S.A.-Rue de l’Industrie, 8-B- 1400 Nivelles-Belgium).

##### Measurement of triglycerides (TG) level in serum

The TG level was estimated following rat triglycerides enzyme-linked immunosorbent assay kit, (BioSource Europe S.A.-Rue de l’Industrie, 8-C- 1150 NivellesBelgium according to^38^.

##### Measurement of high-density lipoproteins (HDL) in serum

The HDL level was estimatedusing a rat HDLcholesterol enzyme-linked immunosorbent assay kit, (BioSource Europe S.A.-Rue de l’Industrie, 8-A- 1340 Nivelles-Belgium)

##### Measurement of low-density lipoproteins (LDL) in serum

The level of LDL was measured depending on the^39^ method. The LDL was computed as follows: LDL=TC-HDL-TG\5.

Serum very low-density lipoproteins (VLDL) levels: VLDL was estimated as follows: (VLDL= TG\5. 9) according to^35^.

##### Atherogenic indices

The atherogenic indices were computed as recorded by^40^.

Cardiac risk ratio = Total cholesterol/HDL cholesterol.

Atherogenic index of plasma = log (Triglyceride/HDL cholesterol

Atherogenic coefficient = (Total cholesterol - HDL cholesterol) / HDL cholesterol.

##### Measurement of serum C-reactive protein (CRP)

The CRP was estimated by using rat Immuno-enzymometric assay kits, (MonobindInc Lake Forest, Ca 92630, USA), as early recorded by^41^.

##### Measurement of serum creatine phosphokinase muscle/brain (CPK-MB):

The CPK-MB level was calculated by using a commercial kit (CPK-MB Kit, Ultra Group, Egypt) following the protocol of^42^.

##### Measurement of serum cardiac troponin I (cTnI)

The cTnIwas estimated by using a commercial kit (Catalog Number SE120134 collected from Sigma-Aldrich Co.) as previously documented by^43^.

*E.Macro-morphological,Macro-morphometric, and Histopathologicalpictures:* Following the collecting of hematological samples, fixation of animals was performed ventrodorsally then by using a sterile scissor, their thoracic cavities were opened asepticallyto revealthe contents. The sternum was dissected to explore the heart and surrounding pericardial fat from the surrounding lungs and the heart was removed for macro-morphological and macro-morphometrical studies.The pericardial fat in all groups was dissected and their mass was weighed using electrical balance, andthen used for quantitative statistical analysis.

Heart weights are obtained for testing the progress of cardiac hypertrophy. Then the whole body weight (WHW) / body weight (BW ratio) was calculated according to^44^.

The cardiac samples were fixed using buffered neutral formalin (10%), then exposed to dehydration, and cleared in Xylene. Soft melted paraffin was used for infiltrating all specimens then soaked in hard paraffin. Paraffin sections were stained using Harris’s Hematoxylin and Eosin (H and E) stain as a standard staining method to reveal the histological picture, Crossmon’s trichrome stain (for muscle and collagen fibers)^45^.

### Tissue preparation for immunohistochemistry and scoring:

The tissue sections were mounted on slides, and the xylene was used for deparaffinization, then, the sections were re-hydrated, and finally rinsed in phosphate buffer saline (PBS). The sections were soaked in 0.3% hydrogen peroxide in aqueous solution. The sections were then washed in 10% normal rabbit serum to lessen the binding of immunoglobulins^45^. The sections were incubated with antisera containing the primary antibody:

1. 1-Anti-Bax antibody [E63] (ab32503) (1:250), Rabbit monoclonal [E63] to Bax, abcam
2. 2-Anti-Bcl-2 antibody (ab196495) (1:100), Rabbit polyclonal to Bcl-2, abcam

The sections were incubated at room temperature overnight. Excess reagent was thrown off and the slides were washed two times with P.B.S, 5 minutes each. Then, the sections were incubated with diluted biotinylated secondary antibody (1:500). The slides were incubated at room temperature for 30 minutes then were washed two times with P.B.S, 5 minutes each. The sections were kept in a humidified chamber then, incubated at horse radish peroxidase polymer (1:60 diluted by deionized water) for 30 min at room temperature. Diaminobenzidine (DAB) was utilized as chromogen and sections were exposed to incubation (2-4 min) at room temperature. Sections were cleaned, then counterstained with Mayer's hematoxylin, dehydrated, cleared, and then mounted in Canada balsam^46^. All the photomicrographs were taken by using by a high-resolution camera (Canon Powershot G10 14.7 MP Digital Camera with Digital Photo Professional software) attached via a vertical tube to a standard light microscope (Olympus BX 21) at the Department of Histology and Cytology, Zagazig University.

### Scoring

Cardiomyocyte hypertrophy and adipocyte scoring was quantified based on cell count per area (by using H and E stain). While collagen level scoring (by using Crossmon’strichrome stain in order to elevate the accuracy of collagen deposition scoring), and immunohistochemical scoring for the expression of Bax and Bcl2 (by using immune-stained sections) were measured based on staining percentage per area. The scoring system was assessed by examining six slides per each group from the various four groups at 100x magnification then the average scores were calculated. Such system assessed each parameter independently and recorded a score of 0–3. Each sample was taken with a histological score using Abramov’s histological scoring system (Table 1)^47^.

**Table 1:** Showing histological score based on Abramov’s histological scoring system.

Parameters	None	Scant	Moderate	Abundant
Cardiomyocyte hypertrophy score	0	1	2	3
Adipocyte count score	0	1	2	3
Collagen level score	0	1	2	3
Immunohistochemical expression of Bax scoring	0	1	2	3
Immunohistochemical expression of Bcl2 scoring	0	1	2	3

### Determination of Bcl-2 and Bax mRNA according to^48^

Bcl-2 and Bax mRNA Gene Expression Detected by Reverse Transcriptase Polymerase Chain Reaction

The myocardium tissues were rinsed in liquid nitrogen, and immersed and stored in -80°C refrigerator for RNA extraction. Total RNA was isolated from frozen myocardium tissue usingTRNzol RNA Reagent Kit (TIANGEN, Beijing, China) according to manufacture protocol.Purified RNA sample were stored at –80°C. The purity and integrity of total RNA were monitored by spectrophotometer, and electrophoresis was carried out on a denaturing formaldehyde agarose gel and stained with ethidium bromide.300 ng mRNA was reversely transcripted to single-stranded cDNA using Invitrogen M-MLV cDNA kit according to manufacture protocol.

The 2 × Taq PCR Green Mix kit and specific primers for Bcl-2 , Bax and b-actin (control) were utilized for the PCR reaction assayThe initial denaturation step was at 95°C followed by 33 cycles for bcl-2 and 29 cycles for bax for 30 s, annealing for 30 s at 55°C for bcl-2 and 60°C bax, and extension for 30°C seconds at 37°C. The PCR products were electrophoresed on a 2% agarose gel.The photographed gel was analyzed using the gel document system (gel pro version 0.3)software Media Cybermetica USA.The results were expressed as a ratio of target mRNA/b-actin mRNA an external control for each sample.

The oligonucleotide Sequences were as follows:

Bcl2 gene: Forward: 5’GAG TACCTG AAC CGG CAT CT -3’ and Reverse: 5’GAAATC AAA CAG AGG TCG CA -3’.

Bax gene: Forward: 5’TTG CTA CAG GGT TTC ATC CA -3’ and Reverse: 5’GAG TAC CTG AACCGG CAT CT -3’.

### Statistical analysis

The expression of the gathered data in the current perspectives represented as mean ± SD for quantitative variables and analyzed statistically via using the Statistical Package for the Social Sciences (SPSS) program (version 24) (SPSS Inc. Chicago, IL, USA. One-way ANOVA was used for comparing the mean values of more than two groups. The test was counted as significant at *p*-values <0.05. The resulting smaller *p*-value indicated a higher significant value.

For histological scoring, Kruskal-Wallis Test was conducted to examine the differences in cardiomyocyte hypertrophy scoring, adipocytes scoring, collagen level scoring, and immunohistochemical scoring for the expression of Bax and Bcl2 according to the types of groups or treatments taken. Mann Whitney test was performed as a pairwise comparison following a significant Kruskal-Wallis test. P < 0.05 is considered a statistical difference. Statistical analysis was performed by SPSS version 25 (Armonk, NY: IBM Corp) and Graph Pad Prism 8.0.2 (GraphPad Software, Inc).

### Ethical approval

All the experimental procedures were conducted in accordance with the guiding principles for the care and use of research animals and were approved by the Institutional Research Board of Faculty of Medicine, Zagazig University (ZU-IACUC/3/F/249/2022). All animal experiments were complied with the ARRIVE guidelines and were carried out in accordance with the U.K. Animals (Scientific Procedures) Act, 1986 and associated guidelines, EU Directive 2010/63/EU for animal experiments.

### Plant guidelines statement

Experimental research and field studies on plants (chia seeds), including the collection of plant material, are complied with Zagazig University guidelines and legislation. The authors comply with the IUCN Policy Statement on Research Involving Species at Risk of Extinction and the Convention on the Trade in Endangered Species of Wild Fauna and Flora.

## Results

### Macro-morphological results and anthropometric measures

**Figure 1:**
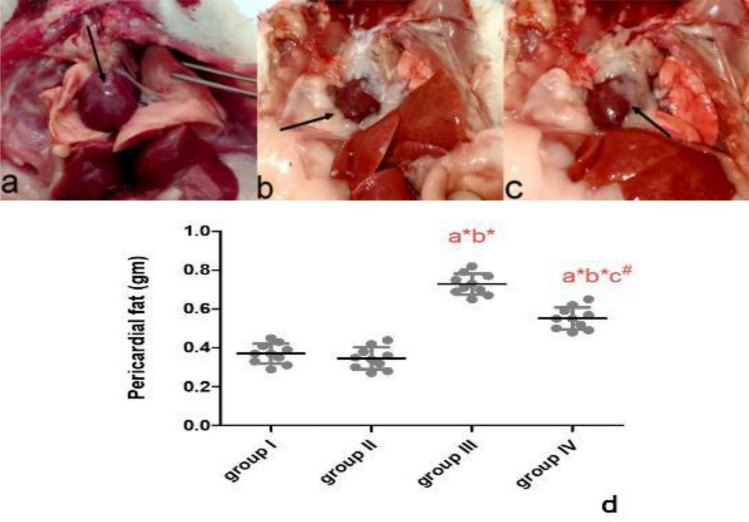
A photo macrograph of rat showing normal amount of pericardial fat surrounding heart representing groups I and II (**a**), high amount of pericardial fat surrounding heart representing high fat diet as group III (**b**) , enhancement and decreases amount of pericardial fat representing high fat diet treated with chia as group IV (**c**) and and Whiskers plot graph shows pericardial fat mass (gm) in all groups, a= vs group I; b= vs group II; c= vs group III; *=significant (P<0.001) and #= significant (P<0.01) (n=10) (**d**).

In the gross anatomical images of hearts, in groups I and group II , amount of pericardial fat surrounding heart are normal (Fig. 1a). Groups (III, IV) accumulated more pericardial fat relative to control rats (groups I, II) (Fig. 1b). However, the amount of fats decreased with chia ingestion (group IV) (Fig. 1c). The HFD increased pericardial fat in group III when compared to groups I and II. While consumption of chia seeds in group IV significantly reversed that effect when compared to group III (Fig. 1d)

**Figure 2:**
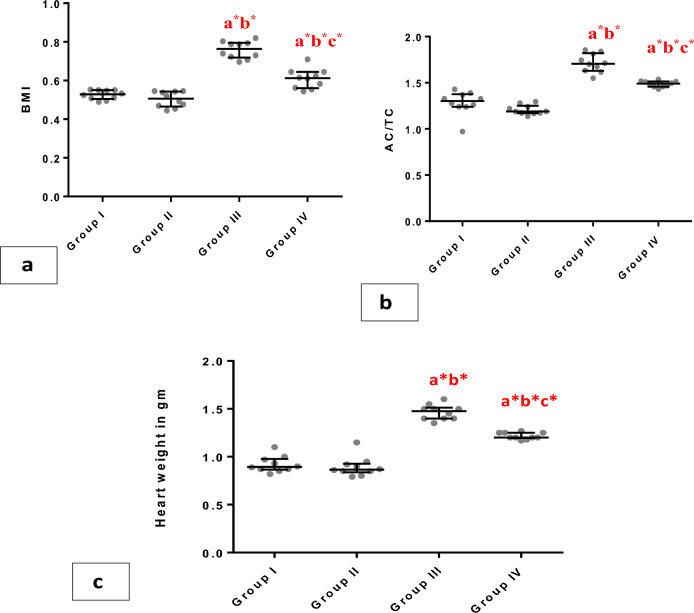
Whiskers plot graphs show anthropometric measures in all groups. (**A**) final body weight (gm), (**B**) abdominal circumference (AC)/ Thoracic circumference (TC) ratio, (**C**) heart weight ingrams.a= vs group I; b= vsgroupII; c= vs group III; *=significant (P<0.001), (n=10).

To assess the development of obesity, we calculated BMI and AC/TC ratio, groups III and IV showed a significant elevation in AC/TC ratio and BMI (p < 0.001) compared to groups I and II. However, these ratios were significantly (p < 0.001) lower in group IV compared to group III (Fig. 2a-c).

There was a clear elevation in heart weight in group III in comparison to groups I, II, and IV (p<0.001). Additionally, the ratio between heart and body weights (H/ BW %) demonstrated no significant difference among groups I, II, III, and IV(0.48±0.01, 0.48±0.0, 050±0.01, and 0.49±0.09 respectively.

### Serum biochemical analysis

**Figure 3:**
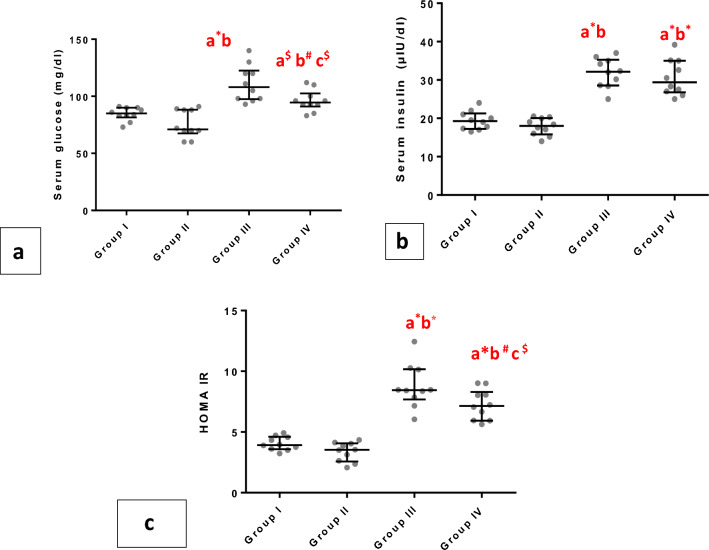
Whiskers plot graphs show glucometabolic parameters in all groups. (**A**) serum glucose (gm/dl), (**B**) serum insulin (μIU/ml), (**C**) homeostasis model assessment of insulin resistance (HOMA). a= vs group I; b= vsgroupII; c= vs group III; $=significant (P<0.05); #=significant (P<0.01); *=significant (P<0.001), (n=10).

As an indication of insulin resistance, the fasting blood glucose and insulin concentrations and HOMA were measured which were noticeably elevated in groups III and VI than in groups I and II (p < 0.001).However; blood glucose and HOMA showed lower levels in group IV (p < 0.05)related to group III (Fig. 3a–c).

**Figure 4:**
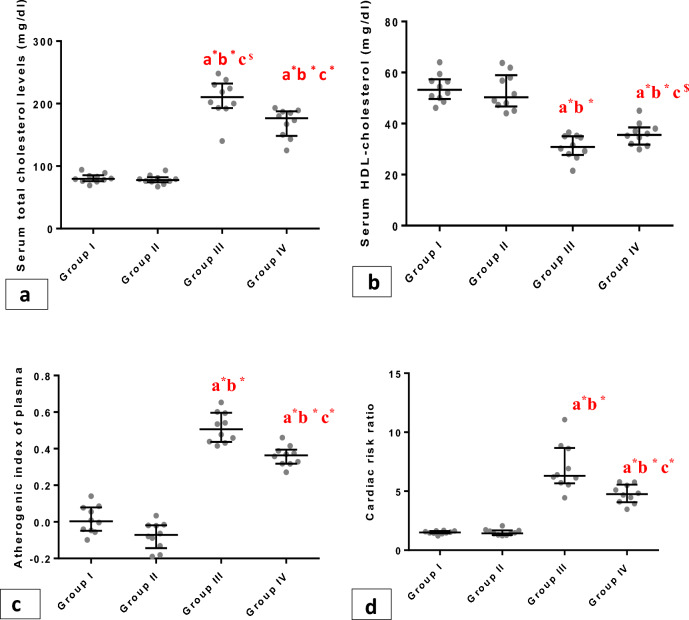
Whiskers plot graphs show serum lipid profile in all groups. (**A**) serum total cholesterol (gm/dl), (**B**) serum high density lipoprotein- cholesterol (HDL-C) (gm/dl), (**C**) Atherogenic index (**D**) cardiac index. a= vs group I; b= vsgroupII; c= vs group III; $=significant (P<0.05); #=significant (P<0.01); *=significant (P<0.001), (n=10).

**Figure 5:**
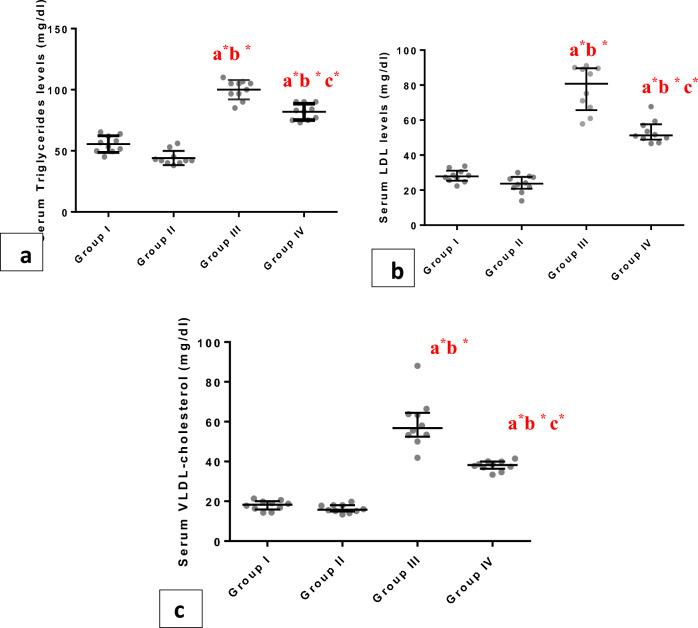
Whiskers plot graphs show serum lipid profile in all groups. (**A**) serum triglyceride (gm/dl), (**B**) serum low density lipoprotein- cholesterol (LDL-C) (gm/dl), (**C**) Serum very low density lipoprotein- cholesterol (LDL-C) (gm/dl). a= vs group I; b= vsgroupII; c= vs group III; *=significant (P<0.001), (n=10).

Regarding biochemical parameters supporting the development of metabolic syndrome, serum TC, LDL,TG, and VLDL levels were estimated and they reported significantly increased values in group III than the group I and group II (p < 0.001), but in group IV the recorded indices were significantly lessened (p < 0.001) when compared with group III. Meanwhile, we reported a significant (P<0.001) reduction in serum HDL in group III compared to groups I and II (Figs. 4, 5a–c). There was a significant increase (p < 0.001) in Atherogenic indices in group III and group IV compared to group I and group II. Group IV exhibited a significant (p < 0.001) decrease in the same indices compared to group III (Fig. 4c).

**Figure 6:**
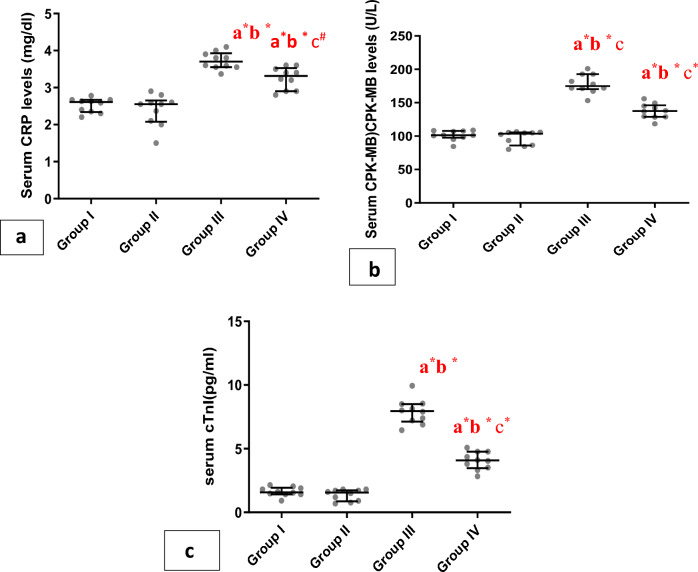
Whiskers plot graphs show serum cardiac inflammatory markers in all groups. (**A**) Serum C-reacive protein (CRP) (μg/dl), (**B**) serum low creatine phosphokinase muscle/brain (CPK-MB) (U/L), (**C**) of Serum cardiac troponin I (cTnI) (pg/ml). a= vs group I; b= vs group II; c= vs group III; *=significant (P<0.001); #=significant (P<0.01), (n=10).

Regarding tissue inflammation as a result of obesity, There was a significant elevation (P<0.001) in serum CRP in group III and group VI compared to group I and group II, chia seeds ingestion significantly (P<0.01) reduced these levels in group IV compared to the group III (Fig. 6a, b, c).

As an indication of cardiac damage, there was a significant (p < 0.001) increase in CK-MB and cTnI in group III and group VI in comparison with group I and group II. Chia ingestion significantly (P<0.001) reduced these levels in group IV compared to group III (Fig. 6a–c).

### Hemodynamic and echocardiography measures

As a metabolic syndrome associated with hypertension, MAP was evaluated and it was recorded a significant (p < 0.001) elevation in group III compared to group I and group II, and these levels were significantly (p < 0.01) reduced in group IV.

**Figure 7:**
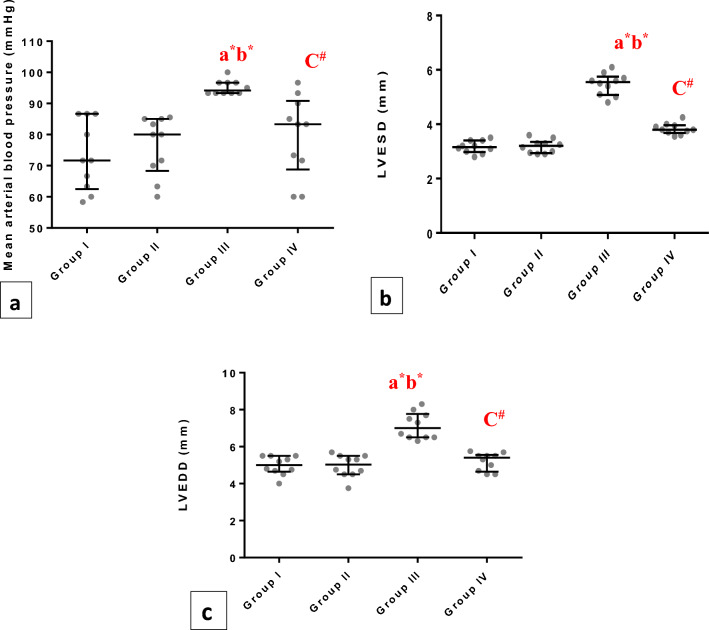
Whiskers plot graphs show Hemodynamic and Echocardiography measures in all groups. (**A**) Mean arterial blood pressure (MAP) (mmHg), (**B**) Left Ventricular End Systolic Diameter (LVESD) (mm), (**C**) Left Ventricular End Systolic Diameter (LVEDD) (mm). a= vs group I; b= vsgroupII; c= vs group III; $=significant (P<0.05); #=significant (P<0.01); *=significant (P<0.001), (n=10).

For assessmentof the effect of obesity on diastolic functions, the present study clarified that LVESD & LVEDD were significantly augmented in group III (p<0.001) when compared to group I and group II.On the other side, these indices were noticeablyimproved by chia ingestion in group IV (Fig. 7a–c).

### Histopathological examination of the heart tissues

**Figure 8:**
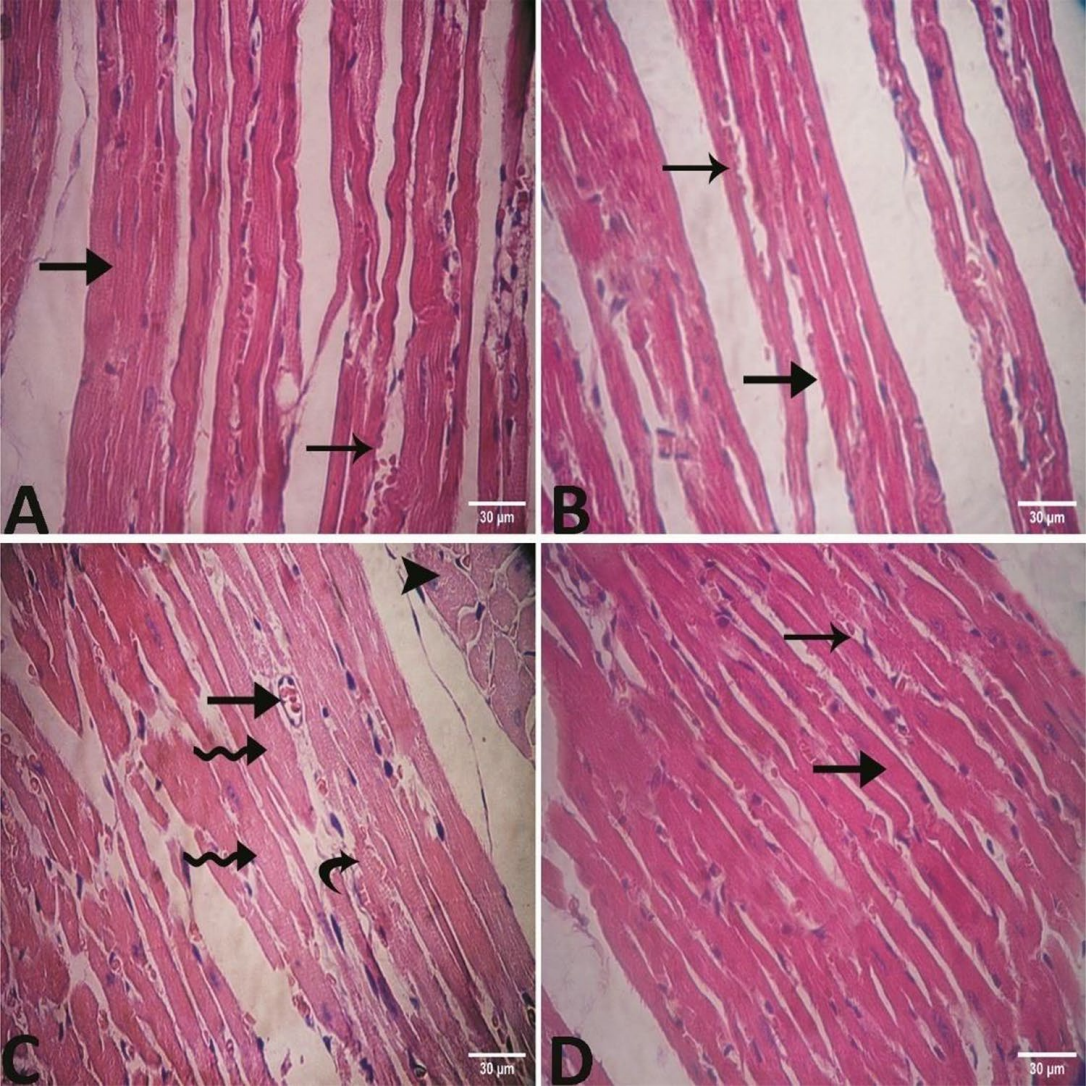
Photomicrograph of the heart (**A**) Group I showing a highly organized myocardium "arrow" with blood vessel "winged arrow". (**B**) Group II showing normal myocardial architecture "arrow" with blood vessel "winged arrow". (**C**) group III displaying structural disorganization, cardiomyocyte hypertrophy "zigzag arrows", slightly degenerated of cardiac myocytes with paler cytoplasm "curved arrow" and pale cross sectional cardiac muscle fibers "arrow head", also showing slightly congested vessels "arrow" (**D**) In group IV, the cardiac muscle showed normal histological structure with branching cardiac muscle bundles "arrow" with blood vessel "winged arrow". Stain: H and E.

The examination of left ventricle sections using a Light microscope revealed these findings: in group I, a highly organized myocardium structure where cardiac muscle fibers appeared cylindrical in shape and extensively branched. They were shorter and usually contained only one nucleus, which was located in the central region of the cell and acidophilic cytoplasm (Fig. 8A).

In group II which fed on a chia diet, the same phenotype was observed and myocardial architecture was normal where cardiac muscle fibers had acidophilic cytoplasm and centrally located nucleus with little collagen fiber between the cardiac muscle bundles (Fig. 8B).

Ingroup III which received a high-fat diet, the high-fat diet induced structural disorganization, accompanied by cardiomyocyte hypertrophy distinguished by a widening in cell surface area, and slightly degenerated cardiac myocytes with paler cytoplasm. There were slightly congested blood capillaries between muscle fibers (Fig. 8C).

In group IV (given a high-fat diet with chia seeds supplementation), the cardiac muscle showed normal histological structure with branched cardiac muscle bundles, acidophilic cytoplasm, single nuclei, and little or no thickening of the matrix around the cardiomyocytesnoticed (Fig. 8D).

**Figure 9:**
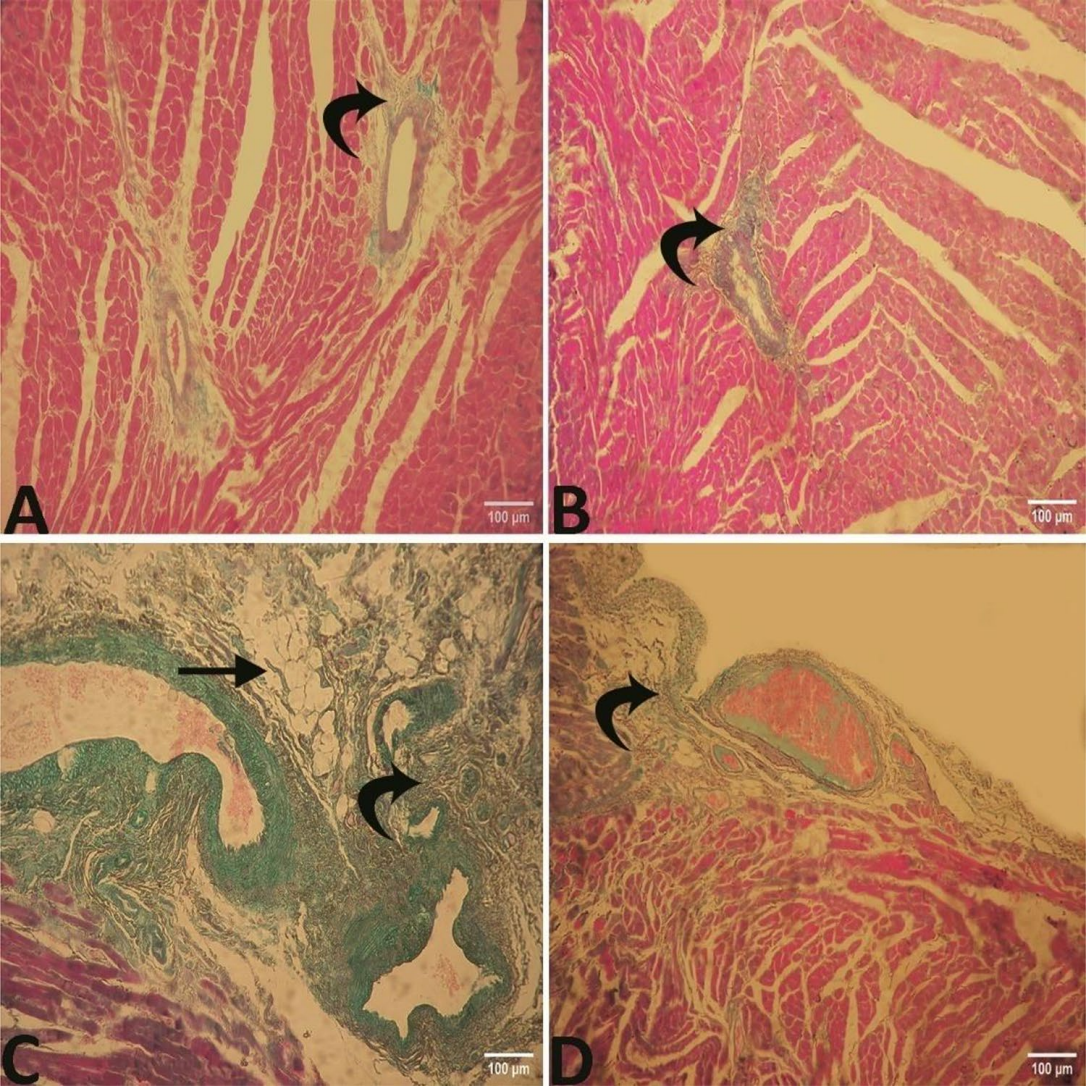
Photomicrograph of the heart (**A**) group I (**B**) group II exhibited minute amount of collagen fibers around the blood vessels "curved arrow" (**C**) group III displayed distinct perivascular fibrosis "curved arrow" also showing some adipocytes "arrow" (**D**) In group IV, the collagen fibers were markedly reduced in quantity "curved arrow". Stain: crossmon'strichrome stain.

By staining the different groups by crossmon's trichrome stain it revealed that group I (Fig. 9A) and group II (Fig. 9B) exhibited minute amounts of collagen fibers around the level of blood vessels. However, in group III, crossmon's trichrome staining displayed interstitial myocardial deposition of collagen fibers and distinct perivascular fibrosis (Fig. 9C). In group IV (Fig. 9D), the collagen fibers were markedly reduced in quantity.

**Figure 10:**
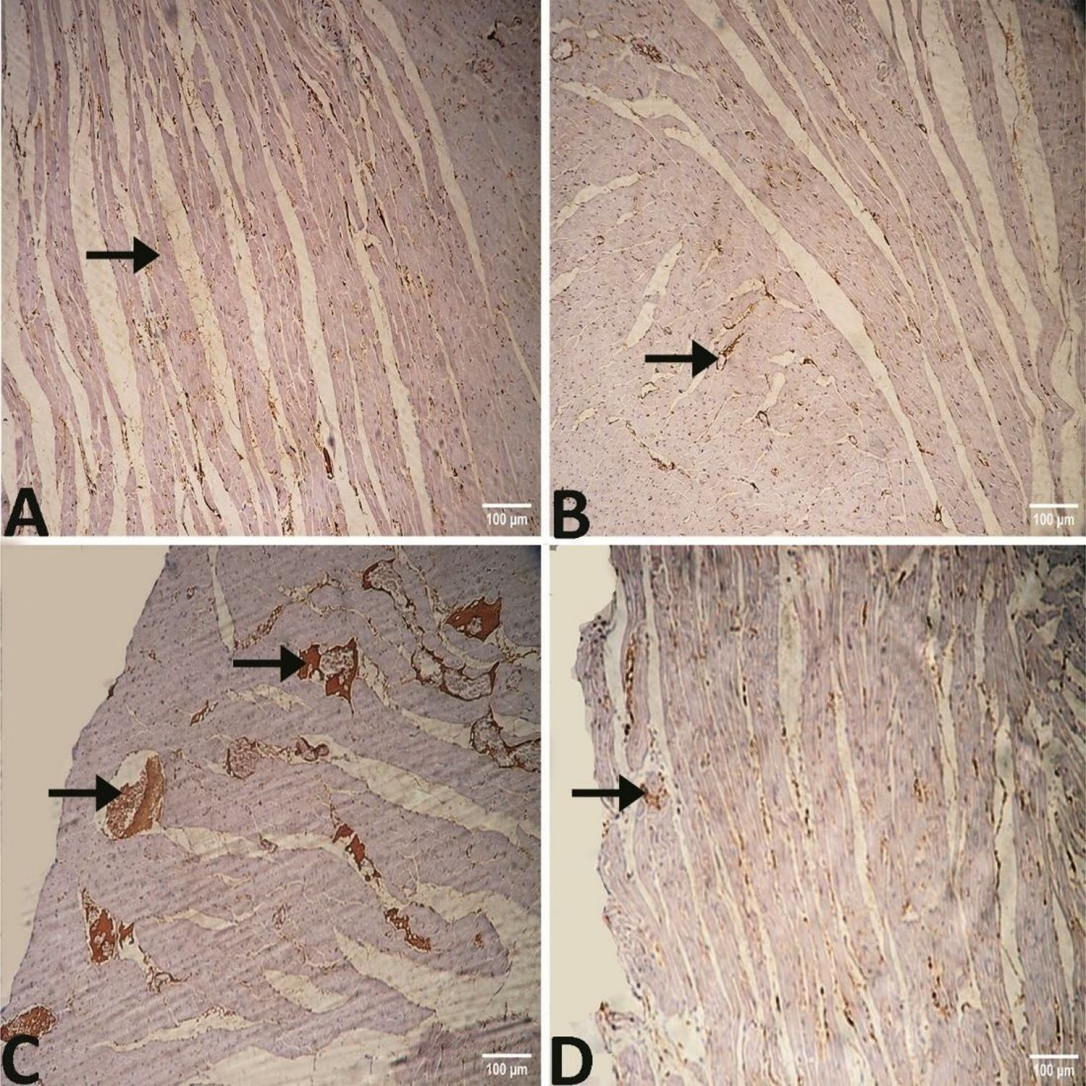
Photomicrograph of the heart immunostainedagainst Bax antibody (**A**) group I showing little reaction "arrow" (**B**) group II showing mild reaction "arrow" (**C**) group III showing significantly higher reaction "arrows" (**D**) group IV showing mild reaction "arrow".

**Figure 11:**
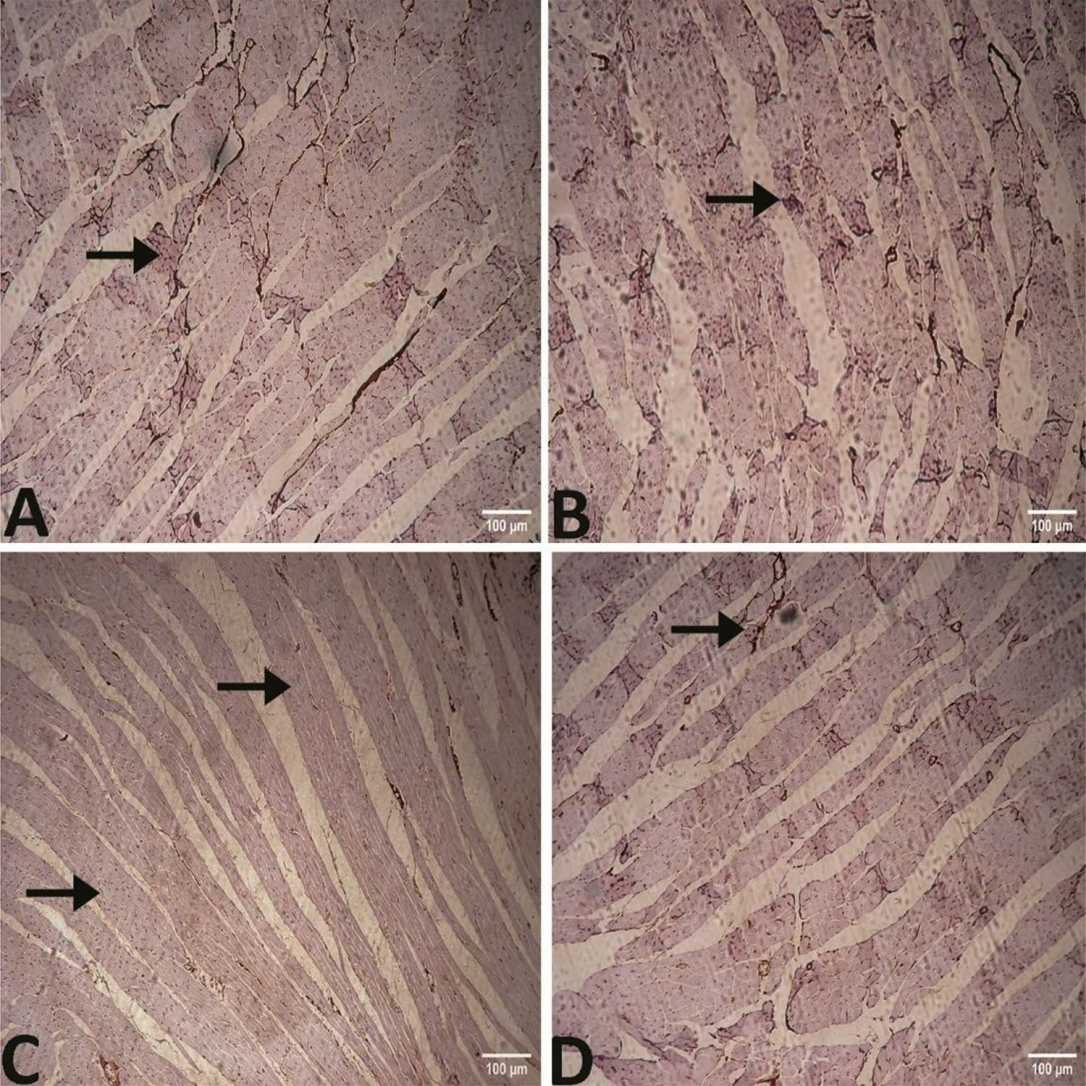
Photomicrograph of the heart immunostainedagainst Bcl-2 antibody (**A**) group I and (B) group II showing significant reaction "arrow" (**C**) group III showing mild reaction "arrow" (**D**) In group IV showing significantly higher reaction "arrow".

In group III, immunohistochemical analysis exhibited that Bcl-2 expression in heart muscle was noticeably lower, but Bax expression was notably elevated. In group IV which was fed on HFD with chia seeds supplementation, Bcl-2 expression was elevated and Bax expression was decreased markedly in the protected group in comparison to groups I and III (Figs. 10 and 11).

### Comparison of the histological scoring of the above-mentioned parameters among the different groups by Kruskal-Wallis Test P < 0.05:

**Figure 12:**
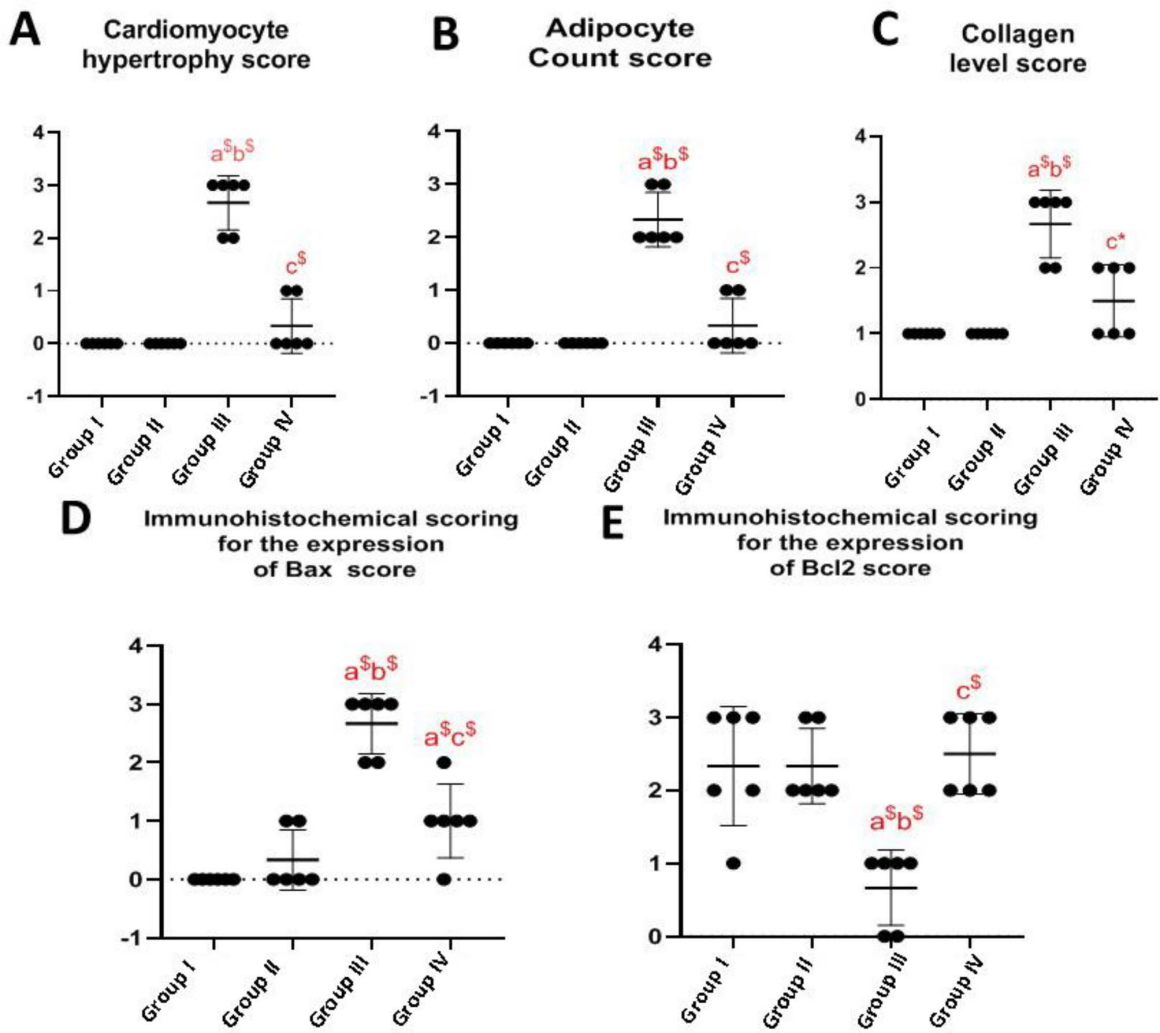
(**A**) Showing a comparison of the cardiomyocyte hypertrophy scoring among the different groups of rats. A minimum of 6 slides were assessed for each group. (**B**) Showing a comparison of the adipocytes count scoring among the different groups of rats. A minimum of 6 slides were assessed for each group. (**C**) Showinga comparison of the collagen level scoring among the different groups of rats. A minimum of 6 slides were assessed for each group. (**D**) Showing a comparison of the immunohistochemical expression of Bax scoring among the different groups of rats. A minimum of 6 slides were assessed for each group. (**E**) showing a comparison of the immunohistochemical expression of Bcl2 scoring among the different groups of rats. A minimum of 6 slides were assessed for each group. a= vs group I; b= vs group II; c= vs group III; *=significant difference (P<0.05); $= highly significant difference (P<0.01).

Kruskal-Wallis Test results showed that group III revealed a significant difference compared with groups I, II, and IV in cardiomyocyte hypertrophy, adipocyte count, collagen level, immunohistochemical expression of Bax scoring, and immunohistochemical expression of Bcl2 scoring. Meanwhile, there was a non-significant difference between groups I and II. Group IV showed a non-significant difference compared to group I in all parameters except the immunohistochemical expression of Bax scoring which showed a highly significance difference. In addition, group IV exhibited a non-significant difference compared to group II in all parameters (Table 2, Fig. 12).

**Table 2:** Showing results of histological scoring.

	Group I	Group II	Group III	Group IV	*p* Value
Mean ranks of cardiomyocyte hypertrophy score	8.5	8.5	21.5	11.5	0.0002^$^
Pairwise comparisons^#^					
Group I	0	0^NS^	− 13$	− 3^NS^	
Group II		0	− 13$	− 3^NS^	
Group III			0	10$	
Group IV				0	
Mean ranks of adipocyte count score	8.5	8.5	21.5	11.5	0.0002^$^
Pairwise comparisons^#^					
Group I	0	0^NS^	− 13$	− 3^NS^	
Group II		0	− 13$	− 3^NS^	
Group III			0	10$	
Group IV				0	
Mean ranks of collagen level score	8	8	21	13	0.0004^$^
Pairwise comparisons^#^					
Group I	0	0^NS^	− 13$	− 5^NS^	
Group II		0	− 13$	− 5^NS^	
Group III			0	8*	
Group IV				0	
Mean ranks of immunohistochemical scoring for the expression of Bax score	6	8.83	21.33	13.83	0.0003^$^
Pairwise comparisons^#^					
Group I	0	− 2.83^NS^	− 15.33$	− 7.83$	
Group II		0	− 12.5$	− 5^NS^	
Group III			0	7.5$	
Group IV				0	
Mean ranks of immunohistochemical scoring for the expression Bcl2 score	15.08	14.83	3.83	16.25	0.004^$^
Pairwise comparisons^#^					
Group I	0	0.25^NS^	11.25$	− 1.17^NS^	
Group II		0	11$	− 1.42^NS^	
Group III			0	− 12.42$	
Group IV				0	

^#^Mannwhitney comparisons test. *Significant difference *p* < 0.05; $ highly significant *p* < 0.01 difference; ns: non-significant difference.

### Findings of Bcl-2 and Bax mRNA

**Figure 13:**
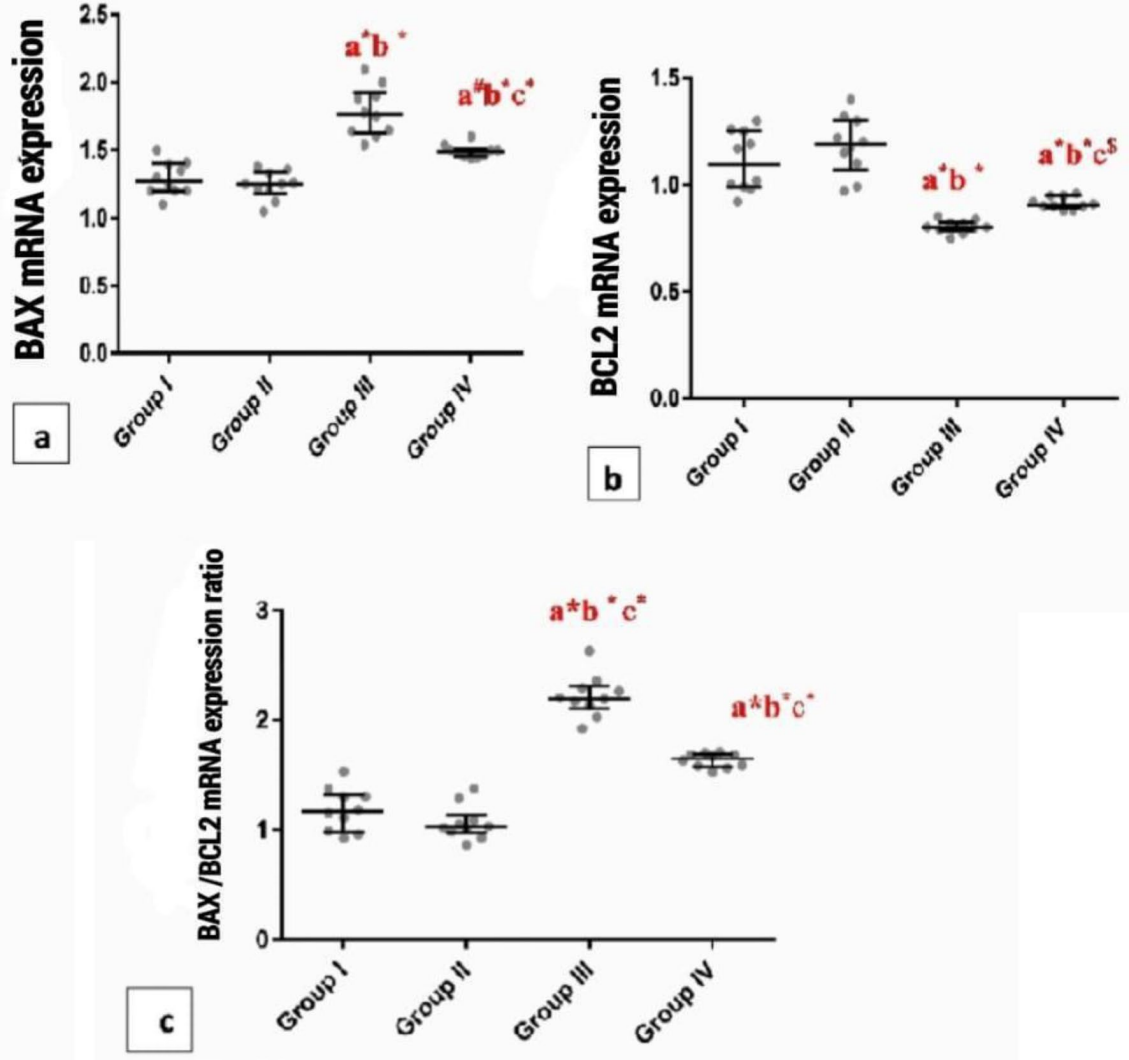
Whiskers plot graphs show cardiac injury markers mRNA expression in all groups. (**A**) Cardiac BAX mRNA expression, (**B**) Cardiac BCL2 mRNA expression, (**C**) Cardiac BAX/BCL2 mRNA expression ratio. a= vs group I; b= vs group II; c= vs group III; $=significant (P<0.05); #=significant (P<0.01); *=significant (P<0.001), (n=10).

HFD had been accompanied by clearly reduced myocardial anti-apoptotic Bcl-2 expression, nd increased pro-apoptotic Bax-expression in comparison to group I (P < 0.001). Additionally, the myocardial Bax/Bcl-2 ratio was significantly higher (P < 0.001) in group III compared with group I. However, chia seed ingestion (group IV) revealed a significant (P < 0.001) modulation in these results (Fig. 13).

## Discussion

Even though the etiology of obesity varies with environmental, genetic, and lifestyle causes, it is greatly linked to co-morbidities like diabetes, cardiovascular diseases, cancer, hypertension, and sleep disorders^49–52^.The link between heart disease and obesity is multifaceted including direct and indirect mechanisms controlled by obesity-associated co-morbidities^53,54^.Chia seeds have an augmented concentration of dietary fibers; hence, it has been utilized for the loss of weight and improves lipid picture and blood glucose^55^. They are rich in proteins, fatty acids, minerals, and antioxidants that are healthy for heart^56^. Therefore, the goal of the present report was to assess the cardiovascular protective influence of chia seed supplementation in high-fat diet-induced obesity.

To assess obesity development and its risk factors, researchers use animal models for diet-induced obesity, since these models imitate human obesity compared to genetic models^57^.

BMI is used for estimating the obesity extension; meanwhile, it reveals no information on the distribution of fat, which is highly essential in cardiovascular risk^58^. Therefore, other clinical assessments (e.g., abdominal circumference) have been used with the goal of identifying central obesity^4^. According to our present findings, we report a clear elevation in BMI and AC/TC ratio in HF-supplemented diet. Also, ingestion of a high-fat diet resulted in the following consequences including elevated levels of fasting serum glucose, increased HOMA-IR index, in addition to elevated fasting levels of serum TG, LDL, TC, VLDL, cardiac risk ratio, atherogenic coefficient, and the atherogenic index, plus a clear reduction in HDL levels. It is opined that rats consuming a high-fat diet are more vulnerable to diabetes and accordingly have insulin resistance. Previous studies verified our findings and recorded that the increased fasting blood glucose level and higher HOMA-IR index, strongly suggest the occurrence of metabolic syndrome^59,60^. Moreover,^61^ mentioned that feeding an enriched diet in high-fat for a period of 10 weeks augmented the level of plasma glucose. In the current study, the elevation of TG could be attributed to increased storage of energy as documented by^62^. Also, the elevated level of cholesterol and the reduction in the level of HDL cholesterol resulted in elevated serum atherogenic coefficient as confirmed by^40^. In line with an earlier study^63^, reported that a high-fat diet produced higher levels of cholesterol, triglycerides, LDL, and VLDL. The main attention is devoted to the ingestion of chia seeds (15%) in group IV which resulted in a clear reduction in BMI and AC/TC ratios and a clear reduction in fasting serum levels of TC, glucose, TG, VLDL, LDL, and HOMA-IR, Cardiac risk ratio, atherogenic coefficient and atherogenic index besides an increase in HDL levels. Such ameliorations could be dominated by the richness of chia seeds in antioxidants and essential fatty acids that could modulate the alterations induced by consuming a high-fat diet.

These outcomes were supported by^15,56,64^who reported that chia seeds are a rich source of dietary fiber that promotes weight loss, improves lipid profile and blood glucose, and reduces blood pressure^15,56,64^. These seeds are rich in 34–36% fibers, which is considered higher compared to other cereals or dried fruit^65,66^, and fatty acids, alpha-linolenic acid (ALA),with a higher ratio^67^, which are useful for controlling metabolic diseases^68^.

In concordance with a previous study^69^, revealed noticeable weight loss by involving chia seeds in bread with a decrease of 500 Kcal daily from the normal diet^70^ documented a significant body weight loss in a group of people eating a hypocaloric diet of a reduction (25% Kcal)from the daily energy need and he attributed this reduction in body weight to chia seed intervention. However^71^, revealed the significance of chia seeds in controlling weight gain. Also^72^ revealed that chia could enhance the reduction of waist circumference. Concurrently^73^, evaluated glycemic responses of chia seeds via lowering blood glucose concentration in healthy individuals. Moreover, other studies were conducted on Wistar rats that received chia seeds and oil and demonstrated a potential impact of lessening TG and elevating HDL^74,75^. Likewise^18,76^, reported that uptake of chia seed in humans induced a significant decline in TG and LDL, while levels of HDL augmented without any side impacts.

Despite the significant reduction in metabolic parameters in group IV, its insulin level, and atherogenic index were still higher than those of control groups, this can be explained by the supplementation of chia seeds was not accompanied by modification of the ingested diet or life style all through the study duration, moreover, these data might be attributed to the dose of chia seeds ingested or the study duration

Ingestion of HFD in the present report induced a noticeable increase in MAP. However, chia intake treatment modulated this alteration. The development of obesity-induced hypertension could be returned to over-activation of the sympathetic nervous system producing hypertension in obese humans and animal models as reported by^77–79^ assumed the mechanism of action of chia seeds which may involve the presence of peptides acting as angiotensin-converting enzyme inhibitors, and thus improve the blood pressure as reported in type 2 diabetes patients. The bioactive peptides with low molecular weight generated from the hydrolysis of chia seed proteins can suppress the activity of ACE^80^.

Previous studies addressed the beneficial components in chia seeds that exert their potential effect on the physiological status and on cardiac health. They are rich in high amounts of insoluble fiber which has been reported to enhance BP more than soluble fiber^74,81^. The components of chia seeds are helpful in cardiovascular disease by lessening blood pressure, cholesterol, aggregation of platelet, and oxidation^25^. Chia is rich in a mixture of essential fatty acids, in a high omega-3 to omega-6 ratio^20,82^, which is associated with lower BP and cardiovascular disease risk^83,84^, and the mineral content for a 100 g carries 335 mg, 407 mg, and 631 mg of Mg, K, and Ca respectively^46^. Both K^85^ and Mg^86^ are opined as strong inhibitors of contraction of vascular smooth muscle and as vasodilators that can lessen BP; Ca is verified to enhance lower BP in hypertensive patients^87,88^.

Echocardiography examination to evaluate cardiac function rats showed a clear increase in LVESD and LVESD in comparison to group I. Our outcomes were in line with previous observations that found the long-term receiving of HFD caused cardiac fibrosis and dysfunction of LV in rodents^89,90^.

Obesity is distinguished by low-grade chronic inflammation with over-expression of pro-inflammatory adipokines in adipose tissue^91,92^. The increased circulatory levels of these inflammatory mediators have been associated with hepatocyte stimulation to produce CRP^92^. In addition, elevated levels of CRP are predictive in some cardiac diseases like unstable angina and myocardial infarction^93,94^. Also, high CRP levels are associated with increased coronary heart disease and overall mortality as reported by^95^.

Similarly^96^, revealed that Wistar rats that received high carbohydrate and high-fat diets had a CRP amount doubled in comparison to the rats that received the same diet and enriched in 5% chia seeds. Further works are recommended to elucidate the mechanism of action of chia seeds with considering inflammatory status. On the other side, these data could indicate an anti-inflammatory condition related to the elevated alpha-linolenic acid (ALA) contents detected in chia seeds^96^.

Our results demonstrated that ingestion of HFD caused significantly elevated CK-MB as well as increased troponin-T levels. However, chia seeds intake significantly ameliorated these levels. These findings were supported by^97^ who mentioned that the release of such markers indicated impaired membrane permeability and cell death in many tissues of the body including the heart. Few studies showed the link between obesity and the occurrence of myocardial damage, including coronary artery diseases. Another study supported our findings and suggested that obesity is in positive association with subclinical myocardial injury, following the National Health and Nutrition Examination Survey (NHASNES) III^98^.

The negative effect of HFD on the heart could be returned to the occurrence of several mechanisms including oxidative damage and inflammation generated by mitochondria dysfunction, cardiac steatosis, altered adipokinespicture, and hyperglycemia. All are the major events that can cause cardiac failure as reported by^99,100^. However, the inflammatory cytokines derived from adipose tissue and those generated locally from the circulating macrophages and monocytes, in response to heart and oxidative damage, promote heart inflammation. Consequently, ROS can damage the cardiac cells and produce hypertrophy, inflammation, and apoptosis by stimulating various damaging pathways (i.e. NF-κB,MAPKs, TGF-β1/Smad2/3, JNK,ASK1, and ANGII)^101,102^.

Previous reports recorded that measures of obesity, such as body mass index, could be accompanied by higher cTn levels in the general public^103,104^. Another study reported significantly elevated serum levels of Troponin-1 and CK-MB in rats that received HFD^105^.

On the tissue level, the hearts in rats fed a standard control diet showed normal histological structure and were well vascularized. Similar results were documented by^106^ report. Also, we reported the occurrence of structural disorganization, slight degeneration, and paler cytoplasm in the supplemented group in high-fat. Similarly^107^, reported that a high-fat diet caused collagen deposits in spaces between cells, cardiomyocytes disorganization, adipose tissue deposits under the pericardium, around vessels, and between cardiomyocytes, interstitial edema, associated with the accumulation of infiltrating cells suggesting cardiomyopathy. The observed cardiac hypertrophy could be induced as a primary step in the sequence of adaptive responses of the cardiac tissue to stress induced by a high number of pathological and physiological conditionsas reported by^108^. Supplementation in chia seeds regenerated the tissue and markedly enhanced these alterations. The same findings were recorded by^106^ in rats.

Based on the findings of Bcl-2 expression, the current study revealed significantly lower expression in heart muscle in the HFD group, meanwhile, Bax expression was significantly higher, suggesting activation of apoptotic processes. It is suggested that myocardial apoptosis can commence by ingestion of a high-fat diet and accumulation of fatty acid metabolites, myocardial, and activation of inflammatory processes, and cardiac insufficiency^109^. Also, in line with another report, Bax can antagonizeBcl-2 function and control the Bcl-2 ability for prolonging the survival of cells as mentioned by^110^. On the other side, dietary intervention in chia seeds produced significant Bcl-2 expression reflecting the potential antioxidant and anti-inflammatory properties.

Apoptosis is seldom in normal myocardium; meanwhile, it can be activated in both chronic and acute heart pathological conditions^111^. Apoptotic signaling produces apoptosis, via three complex pathways including the mitochondrial-mediated pathway, which included Bcl-2 associated X (Bax) protein, B-cell leukemia/lymphoma-2 (Bcl-2) family, and the Bcl-2,which is opined to be essential in controlling apoptosis^112,113^. The ability of anti-apoptotic Bcl-2 to suppress the activity of proapoptotic Bax was dependent on the ratio of Bax to Bcl-2 and on the expression of protein levels^48^. The ratio of Bax to Bcl-2 expression represents a cell death switch, which determines the survival or death of cells in response to an apoptotic stimulus; an increased in this ratio decreases in the cellular resistance to apoptotic stimuli, leading to increased death of cells^114^. Here in, high fat diet increased the pro-apoptotic Bax-to-Bcl-2 gene ratio suggesting occurrence of apoptosis in groups that received high-fat diets, however, the dietary uptake of chia seeds (15%) attenuate this alteration where it elevates the cell resistance via its antioxidant components.

Outcomes of this report reveal that HFD-induced obesity is a risk factor for cardiovascular disease and chia seeds (15% of diet weight) could be a good candidate for cardiovascular system protection.

### Supplementary Information

Supplementary Information.

## Data Availability

All data generated or analyzed during this study are included in this published article and its supplementary information files.

## References

[1] Maric I, Krieger JP, van der Velden P, Börchers S, Asker M, Vujicic M, WernstedtAsterholm I, Skibicka KP. (2022). Sex and species differences in the development of diet-induced obesity and metabolic disturbances in rodents. Front. Nutr..

[2] Barroso TA, Marins LB, Alves R, Gonçalves ACS, Barroso SG (2017). Rocha GDS Association of central obesity with the incidence of cardiovascular diseases and risk factors. Int. J. Cardiovasc. Sci..

[3] Csige, I., Ujvárosy, D., Szabó, Z., Lőrincz, I., Paragh, G., Harangi, M., & Somodi, S. (2018). The impact of obesity on the cardiovascular system. *J. Diabetes Res*. 12 pages. (2018). 10.1155/2018/3407306 PMC6247580 30525052

[4] Csige I, Ujvarosy D, Szabo Z, Lőrincz I, Paragh G, Harangi M, Somodi S (2018). The impact of obesity on the cardiovascular system. J. Diabetes Res..

[5] McGill HC, McMahan CA, Herderick EE, Zieske AW, Malcom GT, Tracy RE (2002). Obesity accelerates the progression of coronary atherosclerosis in young men. Circulation..

[6] Wilson PW, Dagostino RB, Sullivan L, Parise H (2002). Kannel WB Overweight and obesity as determinants of cardiovascular risk: the Framingham experience. ArchIntern Med..

[7] Alpert MA, Omran J, Bostick BP (2016). Effects of obesity on cardiovascular hemodynamics, cardiac morphology, and ventricular function. Curr. Obes. Rep..

[8] Tedrow UB, Conen D, Ridker PM, Cook NR, Koplan BA, Manson JE, Buring JE, Albert CM (2010). The long- and short-term impact of elevated body mass index on the risk of new atrial fibrillation: the WHS (women’s health study)”. J. Am. College Cardiol..

[9] Wang TJ, Parise H, Levy D, D'Agostino RB, Wolf PA, Vasan RS, Benjamin EJ (2004). Obesity and the risk of new-onset atrial fibrillation. JAMA.

[10] Trivedi P, Yang R, Barouch LA (2008). Decreased p110alpha catalytic activity accompanies increased myocyte apoptosis and cardiac hypertrophy in leptin deficient ob/ob mice. Cell Cycle..

[11] Smith CCT, Yellon DM (2011). Adipocytokines, cardiovascular pathophysiology and myocardial protection. Pharmacol. Therapeut..

[12] Leidy HJ (2014). Increased dietary protein as a dietary strategy to prevent and/or treat obesity. Mo Med..

[13] Vaughan, R. A., Conn, C.A., & Mermier, C.M. Effects of commercially available dietary supplements on resting energy expenditure: A brief report. ISRN Nutrition. 2014: 1-7. 2. (2014). 10.1155/2014/650264 PMC4045300 24967272

[14] Whelton SP, Hyrea AD, Pedersen B, Yi Y, Whelton PK, He J (2005). Effect of dietary fiber intake on blood pressure: a meta-analysis of randomized, controlled clinical trials. J. Hypertension..

[15] Lattimer JM, HaubMD. (2010). Effects of dietary fiber and its components on metabolic health. Nutrients.

[16] Ayaz A, Akyol A, Inan-Eroglu E, KabasakalCetin A, Samur G, Akbiyik F (2017). Chia seed (Salvia Hispanica L.) added yogurt reduces short-term food intake and increases satiety: randomized controlled trial. Nutr. Res. Pract..

[17] Coelho MS, Salas-Mellado MDLM (2014). Chemical characterization of CHIA (Salvia hispanica L.) for use in food products. J. Food Nutrit. Res..

[18] Marcinek K, Krejpcio Z (2017). Chia seeds (Salvia hispanica): health promoting properties and therapeutic applications-a review. RoczPanstwZaklHig.

[19] Malta DC, Andrade SC, Claro RM, Bernal RTI, Monteiro AM (2014). Trends in prevalence of overweight and obesity in adults in 26 Brazilian state capitals and the Federal District from 2006 to 2012. Rev. Bras. Epidemiol..

[20] Ullah R, Nadeem M, Khalique A, Imran M, Mehmood S, Javid A, Hussain J (2016). Nutritional and therapeutic perspectives of Chia (Salvia hispanica L.): A review. J. Food Sci. Technol..

[21] Oliveira-Alves SC, Vendramini-Costa BD, Baú BC, Maróstica MR, Ferreira JPB, Silva AB, Prado MA, Bronze MR (2017). Characterization of phenolic compounds in chia (Salvia hispanica L.) seeds, fiber flour and oil. Food Chem.

[22] Vuksan V, Whitham D, Sievenpiper JL, Jenkins AL, Rogovik AL, Bazinet RP (2007). Supplementation of conventional therapy with the novel grain salba (Salvia hispanica L) improves major and emerging cardiovascular risk factors in Type 2 diabetes. Diabetes Care.

[23] Vuksan V, Jenkins AL, Dias AG, Lee AS, Jovanovski E, Rogovik AL, Hanna A (2010). Reduction in postprandial glucose excursion and prolongation of satiety: possible explanation of the long-term effects of whole grain Salba (Salvia Hispanica L.). Eur. J. Clin. Nutr..

[24] Nieman DC, Gillitt N, Jin F, Henson DA, Kennerly K, Shanely RA, Ore B, Su M, Schwartz S (2012). Chia seed supplementation and disease risk factors in overweight women: a metabolomics investigation. J. Altern. Complement Med..

[25] Khalid W, Arshad MS, Aziz A, Rahim MA, Qaisrani TB, Afzal F, Ali A, Ranjha MMAN, Khalid MZ, Anjum FM (2023). Chia seeds (Salvia hispanica L.): A therapeutic weapon in metabolic disorders. Food Sci. Nutr..

[26] Cano PG, Santacruz A, Trejo FM, Sanz Y (2013). Bifidobacterium CECT 7765 improves metabolic and immunological alterations associated with obesity in high-fat diet-fed mice. Obesity.

[27] Rui Y, Yang S, Chen L-H, Qin L-Q (2018). Wan Z Chia seed supplementation reduces senescence markers in epididymal adipose tissue of high-fat diet-fed SAMP8 mice. J. Med. Food.

[28] Fernandez-Martinez E, Lira-Islas I, Cariño R, Soria-Jasso L, Pérez- Hernández E, Pérez-Hernández N (2019). Dietary chia seeds (Salvia hispanica) improve acute dyslipidemia and steatohepatitis in rats. J. Food Biochem..

[29] Novelli ELB, Diniz YS, Galhardi CM, Ebaid GMX, Rodrigues HG, Mani F, Fernandes AAH, Cicogna A, NovelliFilho J (2007). Anthropometrical parameters and markers of obesity in rats. Lab. Anim..

[30] Sausen G, Vieceli T, Rodrigues CG, Kipper D, Stein AT, Grezzana GB (2018). Central hemodynamic parameters to predict cardiovascular outcomes and mortality among the elderly: protocol for a systematic review. Sao Paulo Med J..

[31] Miller FN, Wiegman DL (1997). Anesthesia-induced alteration of small vessel response to norepinephrine. Eur. J. Pharmacol..

[32] Malinowski M, Proudfoot AG, Eberhart L, Schubert H, Wodarek J, Langholz D, Rausch MK, Timek TA (2018). Large animal model of acute right ventricular failure with functional tricuspid regurgitation. Int. J. Cardiol..

[33] Unal I (2017). Defining an optimal cut-point value in roc analysis: an alternative approach. Comput. Math. Methods Med..

[34] Yang H, Lauv S, Sinclair DA (2006). Nampt/PBEF/ Visfatin: A regulator of mammalian health and longevity. Exp. Geront..

[35] Tietz NW, Cook T, McNiven MA (1995). Clinical Guide to Laboratory Tests.

[36] Temple RC, Clark PM, Hales CN (1992). Measurement of insulin secretion in type II diabetes: problems and pitfalls. Diabetic Med..

[37] Sun G, Bishop J, Khalili S, Vasdev S, Gill V, Pace D, Fitzpatrick D, Randell E, Ya- Xie G, Zhang H (2007). Serum visfatin concentrations are positively correlated with serum triacylglycerols and down-regulated by overfeeding in healthy young men. Am. J. Clin. Nutr..

[38] Fossati P (1982). Principle lab. Clin Chem..

[39] Friedewald WT, Levy RI, Fredrickson DS (1972). Estimation of the concentration of low-density lipoprotein cholesterol in plasma, without use of the preparative ultracentrifuge. Clin. Chem..

[40] Ikewuchi JC, Ikewuchi CC (2009). Alteration of plasma lipid profile and atherogenic indices of cholesterol loaded rats by Tridaxprocumbens Linn: implications for the management of obesity and cardiovascular diseases. Biokemistri.

[41] Ridker PM, Rifai N, Pfeffer MA, Sacks FM, Moye LA, Goldman S, Flaker GC (1998). Braunwald inflammation, pravastatin and the risk of coronary events after myocardial infarction in patients with average cholesterol levels. Circulation.

[42] Cabaniss, C.D. Creatine Kinase. In: Walker, H.K., Hall, W.D., Hurst, J.W., editors. Clinical Methods: The History, Physical, and Laboratory Examinations. 3rd edition. Boston: Butterworths; 1990. Chapter 32. https://www.ncbi.nlm.nih.gov/books/NBK352/21250045

[43] Etievent J, Chocron S, Toubin G, Taberlet C, Alwan K, Clement F, Cordier A, Schipman N, Kantelip J (1995). The use of cardiac troponin I as a marker of peri-operative myocardial ischemia. Ann. Thorac. Surg..

[44] Liang B, Zhao Y, Wang X, Yu X, Li Y, Hui-Yu YH, Su Q, Kang Y, Yang Z (2018). Angiotensin-(1–7) attenuates hypertension and cardiac hypertrophy via modulation of nitric oxide and neurotransmitter levels in the paraventricular nucleus in salt-sensitive hypertensive rats. RSC Adv..

[45] Saber S, Khalil RM, Abdo WS, Nassif D, El-Ahwany E (2019). Olmesartan ameliorates chemically-induced ulcerative colitis in rats via modulating NFκB and Nrf-2/HO-1 signaling crosstalk. Toxicol. Appl. Pharmacol..

[46] Suvarna, K., Layton, C., John, D. Bancroft’s theory and practice of histological techniques. 8th ed., Churchil Livingstone, New York, London, Ch. 6: 73-96, Ch. 10: 126-139, Ch. 19: 337- 395. (2018).

[47] Abramov Y, Golden B, Sullivan M, Botros SM, Miller JJR, Alshahrour A (2007). Histologic characterization of vaginal vs. abdominal surgical wound healing in a rabbit model. Wound Repair Regen..

[48] Wang Y, Zhang H, Chai F, Liu X, Berk M (2014). The effects of escitalopram on myocardial apoptosis and the expression of Bax and Bcl-2 during myocardial ischemia/reperfusion in a model of rats with depression. BMC Psychiatry.

[49] Zhang Y, Liu J, Yao J, Ji G, Qian L, Wang J, Zhang G, Tian J, Zhang YE, Gold MS, Liu Y (2014). Obesity: pathophysiology and intervention. Nutrients..

[50] Poirier P (2006). Obesity and cardiovascular disease: pathophysiology, evaluation, and effect of weight loss: an update of the 1997 american heart association scientific statement on obesity and heart disease from the Obesity Committee of the Council on Nutrition, Physical Activity, and Metabolism. Circulation..

[51] Vassallo J (2007). Pathogenesis of obesity. J. Malta Coll. Pharm. Pract..

[52] Litwin SE (2008). Which measures of obesity best predict cardiovascular risk?. J. Am. Coll. Cardiol..

[53] Bays, H.E., Taub, P.R., Epstein, E., Michos, E.D., Ferraro, R.A., Bailey, A.L., Kelli, H.M., Ferdinand, K.C., Echols, M.R., Weintraub, H., Bostrom, J., Johnson, H.M., Hoppe, K.K., Shapiro, M.D., German, C.A., Virani. S.S., Hussain, A., Ballantyne, C.M., Agha, A.M., Toth. P. P. Ten things to know about ten cardiovascular disease risk factors. *Am. J. Prev. Cardiol. *2021;5 (2021). 10.1016/j.ajpc.2021.100149 PMC8315386 34327491

[54] Bays, H.E., McCarthy, W., Burridge, K., Tondt, J., Karjoo, S., Christensen, S., Ng, J., Golden, A., Davisson, L., Richardson, L. Obesity Algorithm eBook, presented by the Obesity Medicine Association. (2022).

[55] Toscano LT, Toscano LT, Tavares RL, da Silva CSO, Silva AS (2015). Chia induces clinically discrete weight loss and improves lipid profile only in altered previous values. Nutr. Hosp..

[56] US Department of Agriculture (USDA) Food surveys research group, What’s In The Foods You Eat Search Tool, 2017- 2018. Beltsville, MD. Available at: http://www.ars.usda.gov/ba/bhnrc/fsrg (accessed 30 December 2019).

[57] White, P.A.S., Cercaro, L.M., Araújo, J.M.D., Souza, L.A., Soares, A.F., Barbosa, A.P.O., Ana, P.O.R., Neto, J.M., Marçal, A.C., Machado, U,F., Camargo, E.A., Santos, M.R.V., Brito, L.C. Modelo de obesidadeinduzidapordietahiperlipídica e associada à resistência à ação da insulina e intolerância à glicose. Arq. Bras. Endocrinol. Metabol. (2013). 10.1590/s0004-27302013000500002 23896799

[58] Zeller M, Steg PG, Ravisy J, Lorgis L, Laurent Y, Sicard P, Janin-Manificat L, Beer J-C, Makki H, Lagrost A-C, Rochette L, Cottin Y (2008). Relation between body mass index, waist circumference, and death after acute myocardial infarction”. Circulation.

[59] Drong AW, Lindgren CM, McCarthy MI (2012). The genetic and epigenetic basis of type 2 diabetes and obesity. Clin. Pharmacol. Ther..

[60] De Almeida AR, Monte-Alegre S, Zanini MB, Souza AL, Etchebehere M, Gontijo JA (2014). Association between prehypertension, metabolic and inflammatory markers decreased adiponectin and enhanced insulinemia in obese subjects. Nutr. Metab. (Lond.).

[61] Janga E, ChoibJunge U, Kimid M, Kimb H, Jeonb S, Shinb S, Seonge C, Leef M (2008). Beneficial effects of curcumin on hyperlipidemia and insulin resistance in high fat diet hamsters. Metab. Clin. Exp..

[62] Chaing S, Baumann CA, Kanzaki M, Thurmond DC, Watson RT, Neudauer CL, Macara LG, Pessin JE, Saltiel AR (2001). Insulin-stimulated GlUT4 translocation requires the CAP-dependent activation TC10. Nature.

[63] Vincent AM, Hinder LM, Pop-Busui R, Feldman EL (2009). Hyperlipidemia new therapeutic target for diabetic neuropathy. J. Peripheral Nervous Syst..

[64] Babio N, Balanza R, Basulto J, Bullَ M, Salas-Salvadَ J (2010). Dietary fibre: influence on body weight, glycemic control andplasma cholesterol profile. NutrHosp.

[65] Ixtaina V, Nolasco S, Tomas M (2008). Physical properties of chia (Salvia hispanica L.) seeds. Ind. Crops Prod..

[66] Kulczynski B, Kobus-Cisowska J, Taczanowski M, Kmiecik D, Anna Gramza-Michałowska A (2019). The chemical composition and nutritional value of chia seeds—current state of knowledge. Nutrients.

[67] Timilsena YP, Vongsvivut J, Adhikari R, Adhikari B (2017). Physicochemical and thermal characteristics of Australian chia seed oil. Food Chem..

[68] FAO. Joint FAO/WHO expert consultation on fats and fatty acids in human nutrition. WHO, Geneva (2008).

[69] Brissette CE, Jenkins AL, Vuksan V (2013). The effect of Salvia hispanica L seeds on weight loss in overweight and obese individuals with type 2 diabetes mellitus. Can. J. Diabetes.

[70] Vuksan V, Jenkins AL, Brissette C, Choleva L, Jovanovski E, Gibbs AL, Bazinet RP, Au-Yeung F, Zurbau A, Ho HVT, Duvnjak L, Sievenpiper JL, Josse RG, Hanna A (2017). Salba-chia (Salvia hispanica L.) in the treatment of overweight and obese patients with type-2 diabetes: A double-blind randomized controlled trial. Nutrit. Metab. Cardiovasc. Diseases.

[71] Mihafu FD, Kiage BN, Kimanga AN, Okoth JK (2020). Effect of chia seeds (Salvia hispanica) on postprandial glycaemia, body weight and hematological parameters in rats fed a high fat and fructose diet. Int. J. Biol. Chem. Sci..

[72] Guevara-Cruz M, Tovar A, Aguilar-Salinas C, Medina-Vera I, Gil-Zenteno L, Hernandez-Viveros I, López-Romero P, Ordaz-Nava G, Canizales-Quinteros S, Pineda LEG, Torres N (2012). A dietary pattern including nopal, chia seed, soy protein, and oat reduces serum triglycerides and glucose intolerance in patients with metabolic syndrome. J. Nutrit..

[73] Ho H, Lee AS, Jovanovski E, Jenkins AL, Desouza R, Vuksan V (2013). Effect of whole and ground Salba seeds (Salvia Hispanica L.) on postprandial glycemia in healthy volunteers: a randomized controlled, dose-response trial. Eur. J. Clin. Nutr..

[74] Valenzuela R, Barrera C, González-Astorga M, Sanhueza J, Valenzuela A (2014). Alpha linolenic acid (ALA) from rosacanina, sachainchi and chia oils may increase ALA accretion and its conversion into n-3 LCPUFA in diverse tissues of the rat. Food Funct..

[75] Chicco AG, D'Alessandro ME, Hein GJ, Olive ME, Lombardo YB (2009). Dietary chia seed (Salvia hispanica L.) rich in α-linolenic acid improves adiposity and normalizes hypertriacylglycerolaemia and insulin resistance in dyslipaemic rats. Br. J. Nutrit..

[76] Flachs P, Rossmeisl M, Bryhn MK (2008). Cellular and molecular effects of n-3 polyunsaturated fatty acids on adipose tissue biology and metabolism. Clin. Sci..

[77] Hall JE (2003). The kidney, hypertension, and obesity. Hypertension..

[78] Grassi G, Dell’Oro R, Facchini A, QuartiTrevano F, Bolla GB, Mancia G (2004). Effect of central and peripheral body fat distribution on sympathetic and baroreflex function in obese normotensives. J. Hypertens..

[79] Arandomized controlled trial (2021). Alwosais EZM, Al-Ozairi E, Zafar TA, Alkandari S. Chia seed (Salvia hispanica L.) supplementation to the diet of adults with type 2 diabetes improved systolic blood pressure. Nutr. Health.

[80] Segura-Campos M, Salazar-Vega I, Chel-Guerrero L, Betancur-Ancona D (2013). Biological potential of chia (Salvia hispanica L.) protein hydrolysates and their incorporation into functional foods. LWT Food Sci. Technol..

[81] Aljuraiban G, Griep L, Chan Q, DaviglusM L, Stamler J, Horn LV, Elliott P, Frost GS (2015). Total, insoluble and soluble dietary fibre intake in relation to blood pressure: The INTERMAP Study. Br. J. Nutrit..

[82] Bresson J, Flynn A, Heinonen M, Hulshof K, Korhonen H, Lagiou P, Løvik M, Marchelli R, Martin A, Moseley B, Palou A, Przyrembel H, Salminen S, Strain JSJ, Strobel S, Tetens I, van den Berg H, van Loveren H, Verhagen H (2009). Opinion on the safety of ‘chia seeds (Salvia hispanica L.) and ground whole chia seeds’ as a food ingredient. EFSA J..

[83] Takeuchi H, Sakurai C, Noda R, Sekine S, Murano Y, Wanaka K, Kasai M, Watanabe S, Aoyama T, Kondo K (2007). Antihypertensive effect and safety of dietary alpha-linolenic acid in subjects with high-normal blood pressure and mild hypertension. J. Oleo Sci..

[84] West S, Krick A, Klein L, Zhao G, Wojtowicz TF, McGuiness M, Bagshaw DM, Wagner P, Ceballos RM, Holub BJ, Kris-Etherton PM (2010). Effects of diets high in walnuts and flaxseed oil on hemodynamic responses to stress and vascular endothelial function. J. Am. Coll. Nutrit..

[85] Suter P, Sierro C, Vetter W (2002). Nutritional factors in the control of blood pressure and hypertension. Nutrit. Clin. Care.

[86] Sontia B, Touyz RM (2007). Role of magnesium in hypertension. Arch. Biochem. Biophys..

[87] Dickinson H, Mason J, Nicolson D, Campbell F, Beyer FR, Julia VC, Williams B, Ford GA (2006). Lifestyle interventions to reduce raised blood pressure: A systematic review of randomized controlled trials. J. Hypertension.

[88] Wang L, Manson J, Buring J, Lee IM, Sesso HD (2008). Dietary intake of dairy products, calcium, and vitamin D and the risk of hypertension in middle-aged and older women. Hypertension.

[89] Aubin MC, Lajoie C, Clément R, Gosselin H, Calderone A, Perrault LP (2008). Female rats fed a high-fat diet were associated with vascular dysfunction and cardiac fibrosis in the absence of overt obesity and hyperlipidemia: therapeutic potential of resveratrol. J. Pharmacol. Exp. Therapeut..

[90] Qin F, Siwik DA, Luptak I, Hou X, Wang L, Akiko A, Weisbrod RM, Ouchi N, Tu VH, Calamaras TD, Miller EJ, Verbeuren TJ, Walsh K, Cohen RA, Colucci WS (2012). The polyphenols resveratrol and S17834 prevent the structural and functional sequelae of diet-induced metabolic heart disease in mice. Circulation.

[91] Karastergiou K, Mohamed-Ali V (2010). Theautocrine and paracrine roles of adipokines. Mol. Cell Endocrinol..

[92] Ellulu MS, Khazaai H, Rahmat A, Patimah I, Abed Y (2016). Obesity can predict and promote systemic inflammation in healthy adults. Int. J. Cardiol..

[93] Liuzzo G, Biasucci LM, Gallimore JR, Grillo RL, Rebuzzi AG, Pepys MB, Maseri A (1994). The prognostic value of C-reactive protein and serum amyloid A protein in severe unstable angina. N. Engl. J. Med..

[94] Anzai T, Yoshikawa T, Shiraki H, Asakura Y, Akaishi M, Mitamura H, Ogawa S (1997). C-reactive protein as a predictor of infarct expansion and cardiac rupture after a first Q wave acute myocardial infarction. Circulation..

[95] Kuller LH, Tracy RP, Shaten J, Meilahn EN (1996). Relation of C-reactive protein and coronary heart disease in the MRFIT nested case-control study. Am. J. Epidemiol..

[96] Poudyal H, Panchal SK, Waanders J, Ward L, Brown L (2012). Lipid redistribution by α-linolenicacidrich chia seed inhibits stearoyl-CoA desaturase-1 and induces cardiac and hepatic protection in dietinduced obese rats. J. NutrBiochem.

[97] Feriani A, Tir M, Gomez-Caravaca AM, Contreras MM, Talhaoui N, Taamalli A, Segura-Carretero A, Ghazouani L, Mufti A, Tlili N, Allagui MS (2020). HPLC-DAD-ESI-QTOF-MS/MS profiling of Zygophyllum album roots extract and assessment of its cardioprotective effect against deltamethrin-induced myocardial injuries in rat, by suppression of oxidative stress-related inflammation and apoptosis via NF-κB signaling pathway. J. Ethnopharmacol..

[98] Vasim I, Ahmad MI, Mongraw-Chaffin M, Soliman EZ (2019). Association of obesity phenotypes with electrocardiographic subclinical myocardial injury in the general population. Clin. Cardiol..

[99] Dludla PV, Joubert E, Muller CJF, Louw J, Johnson R (2017). Hyperglycemia-induced oxidative stress and heart disease-cardioprotective effects of rooibos flavonoids and phenylpyruvic acid-2-O-β-D-glucoside. Nutr. Metab. (Lond)..

[100] Nakamura M, Sadoshima J (2020). Cardiomyopathy in obesity, insulin resistance and diabetes. J. Physiol..

[101] De Marchi, E., Baldassari, F., Bononi, A., Wieckowski, M.R., Pinton, P. Oxidative stress in cardiovascular diseases and obesity: role of p66Shc and protein kinase C. *Oxidative Med. Cell. Longevity* (2013). 10.1155/2013/564961 PMC3625561 23606925

[102] Tsutsui H, Kinugawa S, Matsushima S (2011). Oxidative stress and heart failure American Journal of Physiology. Heart Circ. Physiol..

[103] de Lemos JA, Drazner MH, Omland T, Ayers CR, Khera A, Rohatgi A, Hashim I, Berry JD, Das SR, Morrow DA, McGuire DK (2010). Association of troponin T detected with a highly sensitive assay and cardiac structure and mortality risk in the general population. JAMA.

[104] Saunders JT, Nambi V, de Lemos JA, Chambless LE, Virani SS, Boerwinkle E, Hoogeveen RC, Liu X, Astor BC, Mosley TH, Folsom AR, Heiss G, Coresh J, Ballantyne CM (2011). Cardiac troponin T measured by a highly sensitive assay predicts coronary heart disease, heart failure, and mortality in the Atherosclerosis Risk in Communities Study. Circulation.

[105] Shatoor AS, AlHumayed S (2021). Astaxanthin Ameliorates high-fat diet-induced cardiac damage and fibrosis by upregulating and activating SIRT1. Saudi J. Biol. Sci..

[106] Mishima MDV, Ladeira LCM, da Silva BP, Toledo RLC, de Oliveira TV, Costa NMB, Martino HSD (2021). Cardioprotective action of chia (Salvia hispanica L.) in ovariectomized rats fed a high fat diet. Food Funct..

[107] Sibouakaz D, Othmani-Mecif K, Fernane A, Taghlit A, Benazzoug Y (2018). Biochemical and ultrastructural cardiac changes induced by high-fat diet in female and male prepubertal rabbits. Anal. Cell Pathol. Amst..

[108] Frey N, Katus HA, Olson EN, Hill JA (2004). Hypertrophy of the heart: a new therapeutic target?. Circulation..

[109] Xu J, Zhu L, Liu H, Li M, Liu Y, Yang F, Pei Z (2018). Thymoquinone reduces cardiac damage caused by hypercholesterolemia in apolipoprotein E-deficient mice. Lipids Health Dis..

[110] Sahraoui A, Dewachter C, de Medina G, Naeije R, Aouichat Bouguerra S, Dewachter L (2016). Myocardial structural and biological anomalies induced by high fat diet in psammomysobesus gerbils. PLoS One..

[111] Favaloro B, Allocati N, Graziano V, DiIlio C, DeLaurenzi V (2012). Role of apoptosis in disease. Aging (Albany NY).

[112] Peterson JM, Bryner RW, Sindler A, Frisbee JC, Alway SE (2008). Mitochondrial apoptotic signaling is elevated in cardiac but not skeletal muscle in the obese Zucker rat and is reduced with aerobic exercise. J. Appl. Physiol. (1985).

[113] Marin-Garcia J, Goldenthal MJ (2008). Mitochondrial centrality in heart failure. Heart Fail Rev..

[114] Vaskivuo TE, Stenback F, Tapanainen JS (2002). Apoptosis and apoptosis-related factors Bcl-2, Bax, tumor necrosis factor-alpha, and NF-kappaB in human endometrial hyperplasia and carcinoma. Cancer.

