# Supplementary material for: Chia seeds ameliorate cardiac disease risk factors via alleviating oxidative stress and inflammation in rats fed high-fat diet
Source: Sci Rep. 2024 Feb 5;14:2940. doi: 10.1038/s41598-023-41370-4 (PMC10844609; doi:10.1038/s41598-023-41370-4)
Supplement: Supplementary file 1 — Supplementary Information. [file 41598_2023_41370_MOESM1_ESM.docx]

**Supplementary figure1**


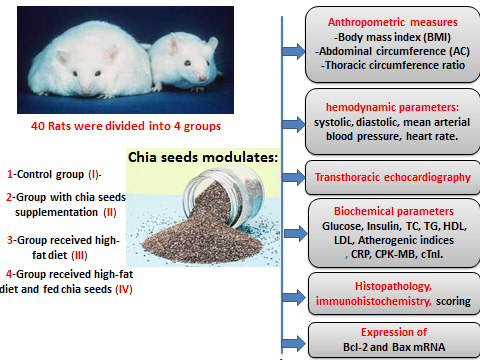


**Figure legand for the supplementary figure1** illustrates the experimental design and the efficacy of chia seeds on cardiovascular health
